# Decreased myelin proteins in brain donors exposed to football-related repetitive head impacts

**DOI:** 10.1093/braincomms/fcad019

**Published:** 2023-03-06

**Authors:** Michael L Alosco, Monica Ly, Sydney Mosaheb, Nicole Saltiel, Madeline Uretsky, Yorghos Tripodis, Brett Martin, Joseph Palmisano, Lisa Delano-Wood, Mark W Bondi, Gaoyuan Meng, Weiming Xia, Sarah Daley, Lee E Goldstein, Douglas I Katz, Brigid Dwyer, Daniel H Daneshvar, Christopher Nowinski, Robert C Cantu, Neil W Kowall, Robert A Stern, Victor E Alvarez, Jesse Mez, Bertrand Russell Huber, Ann C McKee, Thor D Stein

**Affiliations:** Boston University Alzheimer’s Disease Research Center and CTE Center, Department of Neurology, Boston University Chobanian & Avedisian School of Medicine, Boston, MA, USA; Veterans Affairs San Diego Healthcare System, San Diego, CA, USA; Department of Psychiatry, University of California San Diego Health, La Jolla, CA, USA; Boston University Alzheimer’s Disease Research Center and CTE Center, Department of Neurology, Boston University Chobanian & Avedisian School of Medicine, Boston, MA, USA; Boston University Alzheimer’s Disease Research Center and CTE Center, Department of Neurology, Boston University Chobanian & Avedisian School of Medicine, Boston, MA, USA; Boston University Alzheimer’s Disease Research Center and CTE Center, Department of Neurology, Boston University Chobanian & Avedisian School of Medicine, Boston, MA, USA; Department of Biostatistics, Boston University School of Public Health, Boston, MA, USA; Biostatistics and Epidemiology Data Analytics Center, Boston University School of Public Health, Boston, MA, USA; Biostatistics and Epidemiology Data Analytics Center, Boston University School of Public Health, Boston, MA, USA; Veterans Affairs San Diego Healthcare System, San Diego, CA, USA; Department of Psychiatry, University of California San Diego Health, La Jolla, CA, USA; Veterans Affairs San Diego Healthcare System, San Diego, CA, USA; Department of Psychiatry, University of California San Diego Health, La Jolla, CA, USA; VA Bedford Healthcare System, Bedford, MA, USA; VA Bedford Healthcare System, Bedford, MA, USA; Department of Pharmacology and Experimental Therapeutics, Boston University Chobanian & Avedisian School of Medicine, Boston, MA, USA; VA Bedford Healthcare System, Bedford, MA, USA; Department of Pharmacology and Experimental Therapeutics, Boston University Chobanian & Avedisian School of Medicine, Boston, MA, USA; Boston University Alzheimer’s Disease Research Center and CTE Center, Department of Neurology, Boston University Chobanian & Avedisian School of Medicine, Boston, MA, USA; Department of Radiology, Boston University Chobanian & Avedisian School of Medicine, Boston, MA, USA; Departments of Pathology and Laboratory Medicine, Boston University Chobanian & Avedisian School of Medicine, Boston, MA, USA; Department of Psychiatry, Boston University Chobanian & Avedisian School of Medicine, Boston, MA, USA; Departments of Biomedical, Electrical & Computer Engineering, Boston University College of Engineering, Boston, MA, USA; Boston University Alzheimer’s Disease Research Center and CTE Center, Department of Neurology, Boston University Chobanian & Avedisian School of Medicine, Boston, MA, USA; Braintree Rehabilitation Hospital, Braintree, MA, USA; Boston University Alzheimer’s Disease Research Center and CTE Center, Department of Neurology, Boston University Chobanian & Avedisian School of Medicine, Boston, MA, USA; Braintree Rehabilitation Hospital, Braintree, MA, USA; Boston University Alzheimer’s Disease Research Center and CTE Center, Department of Neurology, Boston University Chobanian & Avedisian School of Medicine, Boston, MA, USA; Concussion Legacy Foundation, Boston, MA, USA; Boston University Alzheimer’s Disease Research Center and CTE Center, Department of Neurology, Boston University Chobanian & Avedisian School of Medicine, Boston, MA, USA; Concussion Legacy Foundation, Boston, MA, USA; Department of Neurosurgery, Boston University Chobanian & Avedisian School of Medicine, Boston, MA, USA; Department of Neurosurgery, Emerson Hospital, Concord, MA, USA; Boston University Alzheimer’s Disease Research Center and CTE Center, Department of Neurology, Boston University Chobanian & Avedisian School of Medicine, Boston, MA, USA; Departments of Pathology and Laboratory Medicine, Boston University Chobanian & Avedisian School of Medicine, Boston, MA, USA; VA Boston Healthcare System, U.S. Department of Veteran Affairs, Jamaica Plain, Boston, MA, USA; Boston University Alzheimer’s Disease Research Center and CTE Center, Department of Neurology, Boston University Chobanian & Avedisian School of Medicine, Boston, MA, USA; Department of Neurosurgery, Boston University Chobanian & Avedisian School of Medicine, Boston, MA, USA; Department of Anatomy and Neurobiology, Boston University School of Medicine, Boston, MA, USA; Boston University Alzheimer’s Disease Research Center and CTE Center, Department of Neurology, Boston University Chobanian & Avedisian School of Medicine, Boston, MA, USA; VA Bedford Healthcare System, Bedford, MA, USA; VA Boston Healthcare System, U.S. Department of Veteran Affairs, Jamaica Plain, Boston, MA, USA; National Center for PTSD, VA Boston Healthcare, Jamaica Plain, Boston, MA, USA; Boston University Alzheimer’s Disease Research Center and CTE Center, Department of Neurology, Boston University Chobanian & Avedisian School of Medicine, Boston, MA, USA; Framingham Heart Study, Boston University Chobanian & Avedisian School of Medicine, Boston, MA, USA; Boston University Alzheimer’s Disease Research Center and CTE Center, Department of Neurology, Boston University Chobanian & Avedisian School of Medicine, Boston, MA, USA; VA Boston Healthcare System, U.S. Department of Veteran Affairs, Jamaica Plain, Boston, MA, USA; National Center for PTSD, VA Boston Healthcare, Jamaica Plain, Boston, MA, USA; Boston University Alzheimer’s Disease Research Center and CTE Center, Department of Neurology, Boston University Chobanian & Avedisian School of Medicine, Boston, MA, USA; VA Bedford Healthcare System, Bedford, MA, USA; Departments of Pathology and Laboratory Medicine, Boston University Chobanian & Avedisian School of Medicine, Boston, MA, USA; VA Boston Healthcare System, U.S. Department of Veteran Affairs, Jamaica Plain, Boston, MA, USA; National Center for PTSD, VA Boston Healthcare, Jamaica Plain, Boston, MA, USA; Framingham Heart Study, Boston University Chobanian & Avedisian School of Medicine, Boston, MA, USA; Boston University Alzheimer’s Disease Research Center and CTE Center, Department of Neurology, Boston University Chobanian & Avedisian School of Medicine, Boston, MA, USA; VA Bedford Healthcare System, Bedford, MA, USA; Departments of Pathology and Laboratory Medicine, Boston University Chobanian & Avedisian School of Medicine, Boston, MA, USA; VA Boston Healthcare System, U.S. Department of Veteran Affairs, Jamaica Plain, Boston, MA, USA; Framingham Heart Study, Boston University Chobanian & Avedisian School of Medicine, Boston, MA, USA

**Keywords:** myelin, cerebrovascular disease, chronic traumatic encephalopathy, repetitive head impacts, white matter

## Abstract

American football players and other individuals exposed to repetitive head impacts can exhibit a constellation of later-life cognitive and neuropsychiatric symptoms. While tau-based diseases such as chronic traumatic encephalopathy can underpin certain symptoms, contributions from non-tau pathologies from repetitive head impacts are increasingly recognized. We examined cross-sectional associations between myelin integrity using immunoassays for myelin-associated glycoprotein and proteolipid protein 1 with risk factors and clinical outcomes in brain donors exposed to repetitive head impacts from American football. Immunoassays for myelin-associated glycoprotein and proteolipid protein 1 were conducted on dorsolateral frontal white matter tissue samples of 205 male brain donors. Proxies of exposure to repetitive head impacts included years of exposure and age of first exposure to American football play. Informants completed the Functional Activities Questionnaire, Behavior Rating Inventory of Executive Function-Adult Version (Behavioral Regulation Index), and Barratt Impulsiveness Scale-11. Associations between myelin-associated glycoprotein and proteolipid protein 1 with exposure proxies and clinical scales were tested. Of the 205 male brain donors who played amateur and professional football, the mean age was 67.17 (SD = 16.78), and 75.9% (*n* = 126) were reported by informants to be functionally impaired prior to death. Myelin-associated glycoprotein and proteolipid protein 1 correlated with the ischaemic injury scale score, a global indicator of cerebrovascular disease (*r* = −0.23 and −0.20, respectively, *P*s < 0.01). Chronic traumatic encephalopathy was the most common neurodegenerative disease (*n* = 151, 73.7%). Myelin-associated glycoprotein and proteolipid protein 1 were not associated with chronic traumatic encephalopathy status, but lower proteolipid protein 1 was associated with more severe chronic traumatic encephalopathy (*P* = 0.03). Myelin-associated glycoprotein and proteolipid protein 1 were not associated with other neurodegenerative disease pathologies. More years of football play was associated with lower proteolipid protein 1 [beta = −2.45, 95% confidence interval (CI) [−4.52, −0.38]] and compared with those who played <11 years of football (*n* = 78), those who played 11 or more years (*n* = 128) had lower myelin-associated glycoprotein (mean difference = 46.00, 95% CI [5.32, 86.69]) and proteolipid protein 1 (mean difference = 24.72, 95% CI [2.40, 47.05]). Younger age of first exposure corresponded to lower proteolipid protein 1 (beta = 4.35, 95% CI [0.25, 8.45]). Among brain donors who were aged 50 or older (*n* = 144), lower proteolipid protein 1 (beta = −0.02, 95% CI [−0.047, −0.001]) and myelin-associated glycoprotein (beta = −0.01, 95% CI [−0.03, −0.002]) were associated with higher Functional Activities Questionnaire scores. Lower myelin-associated glycoprotein correlated with higher Barratt Impulsiveness Scale-11 scores (beta = −0.02, 95% CI [−0.04, −0.0003]). Results suggest that decreased myelin may represent a late effect of repetitive head impacts that contributes to the manifestation of cognitive symptoms and impulsivity. Clinical–pathological correlation studies with prospective objective clinical assessments are needed to confirm our findings.

## Teaser

Alosco *et al.* reported more years and younger ages of exposure to football were associated with decreased myelin integrity, using myelin-associated glycoprotein and proteolipid protein-1, among 205 deceased football players. Decreased myelin proteins correlated with functional impairment and impulsivity. Myelin degeneration might be a late effect of repetitive head impacts.

## Graphical Abstract

**Graphical abstract:**
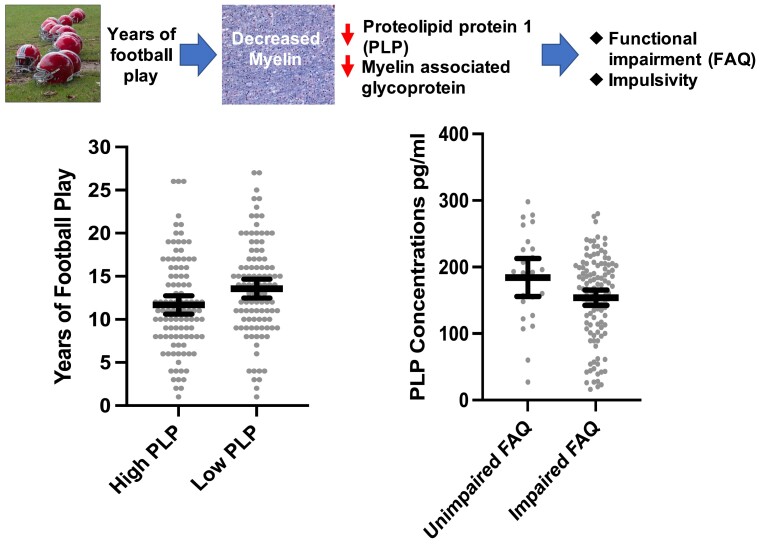

## Introduction

Participation in American football is associated with exposure to repetitive head impacts (RHI) that can lead to symptomatic concussions and asymptomatic sub-concussions.^1–6^ These impacts are associated with cognitive deficits and neuropsychiatric disturbances later in life.^7–11^ Some individuals exposed to RHI go on to develop chronic traumatic encephalopathy (CTE), a progressive neurodegenerative disease characterized by the perivascular deposition of hyper-phosphorylated tau (p-tau) in neurons at the depths of the cerebral sulci.^12,13^ The duration of exposure to RHI (i.e. years of American football play) has been closely tied to risk for CTE.^14,15^ Other aspects of exposure to RHI, such as age of first exposure, may not necessarily confer risk for CTE,^16^ but could increase susceptibility to late-life neurological symptoms and brain alterations.^17–22^ The literature on age of first exposure is mixed, as effects have primarily been observed in older symptomatic former elite football players as opposed to active college-age athletes.^23–28^

At this time, CTE can only be diagnosed at autopsy. Research diagnostic criteria for the clinical syndrome of CTE are known as traumatic encephalopathy syndrome.^29–31^ Core clinical features of traumatic encephalopathy syndrome include cognitive impairment (i.e. deficits in episodic memory and/or executive functioning) and neurobehavioural dysregulation (e.g. explosiveness, impulsivity and emotional lability).^30^ The diverse clinical phenotypes reported among individuals exposed to RHI are likely driven by both tau and non-tau aetiologies.^31^ Exposure to RHI is indeed being increasingly linked with mixed pathologies.^32–36^

White matter injury is the cardinal pathology of acute traumatic brain injury,^37,38^ and exposure to RHI can also lead to white matter injury. Diffusion tensor imaging studies have found white matter changes following just one season of contact sport play, even in the absence of a clinically diagnosed concussion.^39–45^*In vivo* magnetic resonance imaging studies of professional athletes support lasting consequences of exposure to RHI on white matter. Former National Football League players show altered corpus callosum microstructure on diffusion tensor imaging scans^46^ and greater volumes of white matter signal abnormalities on fluid-attenuated inversion recovery (FLAIR)^47^ and T1 scans.^48^ Active professional fighters and retired rugby players also show abnormal diffusivity along several white matter tracts including the corpus callosum on diffusion tensor imaging.^49,50^ These white matter changes have been associated with depression and worse neuropsychological test performance, particularly on measures of psychomotor speed and executive functioning.^46,48,51^ Younger age of first exposure to RHI has also been linked to abnormal diffusivity and reduced volume in the corpus callosum.^17,21^

Autopsy studies have characterized white matter pathologies in brain donors exposed to RHI. In autopsy-confirmed CTE, multifocal axonal varicosities and axonal loss are found at all stages of CTE.^52^ A recent single nuclear RNA sequencing study found a decrease in the number of oligodendrocytes in the dorsolateral frontal white matter in donors with CTE compared with controls.^53^ Additional research by our team that used samples of brain donors with autopsy-confirmed CTE directly linked greater years of American football play to more severe semi-quantitative ratings of white matter rarefaction^54^ as well as to greater volumes of white matter hyperintensities on antemortem FLAIR magnetic resonance imaging.^55^ These white matter pathologies correlated with informant-reported cognitive symptoms.^54,55^ Similar to what is observed in the context of Alzheimer’s disease,^56^ white matter pathologies likely contribute to cognitive, mood and/or behavioural symptoms in people exposed to RHI.

To date, neuropathological studies examining white matter, in general, and among individuals exposed to RHI, in particular, have typically used crude (i.e. semi-quantitative rating scales) and non-specific measurements of white matter. More recently, there has been growing interest in investigating more novel indices such as myelin-associated glycoprotein (MAG) and proteolipid protein 1 (PLP) that may represent the earliest changes to white matter integrity following brain insult. Both MAG and PLP are produced in oligodendrocytes and delivered to the myelin sheath.^57–60^ PLP is distributed throughout the myelin sheath and MAG is located distally from the cell body.^59,60^ Both proteins contribute to the structure, function and maintenance of the myelin and have been used for the study of myelin degeneration in multiple sclerosis, Alzheimer’s disease, and related dementias as they are stable under post-mortem conditions.^61–66^ The literature on MAG and PLP is scarce and somewhat mixed in terms of differences between Alzheimer’s disease and related dementia cases and control groups, but they consistently correlate with cerebrovascular disease.^62,64,65^ The ratio of MAG:PLP has been of focus as an accurate measure of subacute hypoperfusion.^65,67^ Declines in the MAG:PLP ratio reflect hypoperfusion that occurred over the months prior to death because the distal location of MAG renders it vulnerable to ischaemia, whereas PLP remains preserved.^59,60,65,67,68^

Only one study has examined MAG or PLP in the setting of RHI. Bi *et al.*^69^ analysed post-mortem brain tissue from three donors with neuropathologically diagnosed stage III–IV CTE and three controls between ages 73 and 84. They found that both the levels and immunoreactivity of MAG were reduced in CTE brains.^69^ The authors did not investigate PLP or associations with exposure to RHI and clinical functioning. The objective of our study was to use MAG and PLP to characterize myelin integrity and its associated clinical and risk factor correlates in brain donors exposed to RHI. In a large sample of older adults exposed to cumulative RHI through football during their lifetimes and who played at all levels (i.e. youth, high school, college and professional), we examined the associations between MAG and PLP and neurodegenerative and non-neurodegenerative neuropathologies, as well as their associations with proxies of exposure to RHI (i.e. years of play and age of first exposure) and informant-reported cognitive and neuropsychiatric symptoms.

## Materials and methods

### Brain donors and study design

Fresh-frozen brain tissue was obtained from male former American football players who donated their brains to the Veteran’s Affairs-Boston University-Concussion Legacy Foundation brain bank for inclusion in the Understanding Neurologic Injury and Traumatic Encephalopathy study. The methodology for this study has been previously published.^70^ Most brain donations were from next-of-kin who contacted the brain bank near the time of death. Others were referred by medical examiners, recruited by the Concussion Legacy Foundation or participated in the Brain Donation Registry during life. To be eligible, brain donors must have had a history of RHI, such as from contact sport play, military service, physical violence and other sources. The inclusion criteria were recently expanded to include a history of moderate to severe traumatic brain injury. Eligibility for the brain bank was not based on antemortem symptomatic status. Brain donors with poor tissue quality were excluded. For this study, the sample only included former American football players as they make up much of the brain bank and offer some homogeneity in their demographic and exposure to RHI characteristics to appropriately model and interpret associations. Only donations with fresh-frozen tissue available were included to allow for biochemical measurement of MAG and PLP. Institutional review board approval for brain donation, post-mortem clinical record review, interviews with informants, and neuropathological evaluation were obtained through the Boston University Medical Campus institutional review board.

### Neuropathological examination

Brain donations were processed using published protocols.^71,72^ Whole brains were received fresh, on wet ice, and gross pathology was evaluated. Once hemi-sected, one hemisphere was fixed in periodate-lysine-paraformaldehyde and stored at 4°C and the other hemisphere was sectioned coronally and flash-frozen using dry ice. Blocks of tissue from the fixed hemisphere were dissected, embedded in paraffin and cut at 10 µm for immunohistochemistry. Using previously described methods, tissue was stained with Luxol fast blue, haematoxylin and eosin, Bielschowsky silver, and with antibodies for p-tau (Ser202, Thr205), β-amyloid, α-synuclein and phosphorylated transactive response DNA-binding protein of 43 kDa. Neuropathologists blinded to the donors’ clinical and athletic histories used established criteria to diagnose and stage neurodegenerative diseases.^73–79^ National Institute on Aging-Reagan was followed for the diagnosis of Alzheimer’s disease.^74^ Neuropathological diagnosis of CTE was made using criteria defined by the NINDS-NIBIB Consensus Conference.^12,13^ The Understanding Neurologic Injury and Traumatic Encephalopathy study has followed recommendations from the second NINDS-NIBIB Consensus Conference for the neuropathological diagnosis of CTE,^13^ which requires tau inclusions in neurons with variable astrocytic involvement. It states, ‘…the perivascular p-tau aggregates should include neurofibrillary tangles, with or without astrocytes…’^13^ CTE stage was also designated using the McKee staging scheme, classified as low (stage I/II) and high (stage III/IV).^13,52,80^

A modified version of the ischaemic injury scale^81^ was used as a global indicator of cerebrovascular disease and based on the presence of hippocampal sclerosis, infarcts, microinfarcts, microbleeds, laminar necrosis, arteriolosclerosis, atherosclerosis, cerebral amyloid angiopathy and white matter rarefaction. Unlike the original ischaemic injury scale, the cribriform state was not included because it was not rated in more remote cases from our brain bank. Arteriolosclerosis, atherosclerosis, cerebral amyloid angiopathy and white matter rarefaction were rated on a semi-quantitative scale (0 = none, 3 = severe), whereas the remaining pathologies were rated as absent/present. Methods for the detailed assessments of the individual-level cerebrovascular and white matter pathologies have been described elsewhere.^54^ The modified ischaemic injury scale is a summary composite of all the pathologies with a possible range of 0–17.

Neuropathological ratings and diagnoses were conducted by study co-authors (B.R.H., A.C.M. and T.D.S.). They have previously been shown to have very good inter-rater reliability on semi-quantitative rating scales of neuropathology, namely scales of CTE and cerebral amyloid angiopathy severity.^34,80^ While inter-rater reliability across other various individual pathologies is not known, the primary outcome and focus of this study is the quantitative measures of myelin loss (i.e. MAG and PLP) as opposed to the semi-quantitative rating scales.

### Immunoassays for MAG and PLP1

White matter samples were collected from the dorsolateral frontal region of fresh-frozen brain tissue. The dorsolateral frontal cortex was examined because of its known involvement in CTE.^80^ The frozen tissue was weighed and placed on dry ice. Freshly prepared 5 M Guanidine Hydrochloride in Tris-buffered saline (20 mM Tris-HCl, 150 mM NaCl, pH 7.4) containing 1:100 Halt protease inhibitor cocktail (Thermo Fischer Scientific, Waltham, MA) was added to the brain tissue at 5:1 [5 M Guanidine Hydrochloride volume (ml):brain wet weight (g)]; and tissue was homogenized with Qiagen Tissue Lyser LT at 50 Hz for 5 min. The homogenate was diluted 1:100 with phosphate buffered saline (pH = 7.4) and subsequently centrifuged at 17 000 g and 4°C for 15 min. Enzyme-linked immunosorbent assay (ELISA) kits from AVIVA (San Diego, CA) for MAG (OKEH00439) and PLP1 (OKEH00437) were used to measure levels of these proteins in the dorsolateral frontal white matter according to the manufacturer’s protocol. The plates were read on a standard calorimetric plate reader at 450 nm absorbance with background subtraction. Plates for each protein were run in three separate batches with samples repeated as internal controls.

### Retrospective clinical evaluations

Retrospective information about donors’ athletic, military, medical and behavioural history was collected using methods previously described.^70,80,82^ Researchers were blind to the neuropathological analysis and informants were interviewed before receiving the neuropathological results. Informants completed semi-structured phone interviews with clinician researchers and/or online questionnaires and structured phone interviews that included cognitive and neuropsychiatric scales adapted for post-mortem, retrospective evaluation. For this study, we examined the Functional Activities Questionnaire (FAQ),^83^ Behavior Rating Inventory of Executive Function-Adult Version (BRIEF-A) Behavioral Regulation Index (BRI),^84^ and the Barratt Impulsiveness Scale (BIS-11). The FAQ is a 10-item scale of instrumental activities of daily living and scores range from 0 to 30, with higher scores reflecting greater severity of functional impairment. The BRIEF-A is a 75-item measurement of executive function behaviours. Informants rated how often each behaviour had been a problem on a three-point scale (1 = never, 2 = sometimes, 3 = often). BRIEF-A BRI is a measure of an individual’s ability to control impulses and self-monitor. Age-adjusted BRI *T*-scores were examined, and higher scores represent greater dysfunction. The BIS-11 is a 30-item questionnaire designed to assess impulsiveness. BIS-11 scores are ranked on a four-point Likert-type scale, where higher scores reflect more impulsive behaviours. These scales were selected *a priori* to limit the number of analyses and because of their associations with exposure to RHI and representation of the core clinical features of the traumatic encephalopathy syndrome criteria.^30^

The Understanding Neurologic Injury and Traumatic Encephalopathy study has evolved over time with a more standardized, systematic assessment adopted in 2014. Prior to January 2014, only the FAQ was administered, and after 2014, we began to implement scales of cognitive and neuropsychiatric symptoms. Demographics, educational attainment, athletic history (type of sports played, level, position, age of first exposure and duration), military history and traumatic brain injury history were queried during a telephone interview (pre-2014) and/or using an online questionnaire (2014 and on).

### Sample size

Of the brain donors, 277 had available MAG and PLP data at the time of this study. The primary sample size for the present study was reduced to 205 after the exclusion of brain donors in the following iterative order: those who did not play American football (*n* = 56), female sex (*n* = 1) and those who had missing data on primary analytic variables (*n* = 15). Although all of the selected brain donors played football, many also played other contact sports and/or served in the military. For age of first exposure models, one additional brain donor was excluded due to an erroneous age of first exposure. Due to missing data, the sample size was reduced to 166 for models that examined the FAQ, BIS-11 and the BRIEF-A BRI.

### Statistical analyses

Associations between MAG and PLP with demographic (e.g. age, race, education level) and neurodegenerative disease diagnoses and non-neurodegenerative disease (e.g. modified ischaemic injury scale) pathologies were tested using Pearson’s bivariate correlation, chi-square or independent samples *t*-tests. Multivariable linear regressions tested the associations between the exposure to RHI proxies (i.e. years of football play and age of first exposure to football) and MAG and PLP. A separate model was done for each RHI proxy and each ELISA marker. Binary logistic regression models were used to test the association between the exposure proxies and odds for having low MAG and PLP, defined by the sample median split. This was done to facilitate within-sample interpretation of results and effect sizes as well as to account for potential non-linear associations. Previous research showed 11 years of football maximizes sensitivity and specificity for predicting CTE risk.^14^ As a sensitivity analysis, analysis of covariance compared brain donors who played 11 or more years with those who played <11 years on MAG and PLP levels. Estimated marginal mean difference (‘mean diff.’) from the analysis of covariance is reported and refers to the estimated marginal mean difference for the given outcome between groups being compared, and adjusted for the relevant covariates. Note that because years of football and level of play are highly correlated (*r* = 0.74, *P* < 0.001) we did not examine the level of play separately to limit the number of analyses performed.

In separate models, multivariable linear regression analyses tested the association between MAG and PLP and each clinical scale (i.e. FAQ, BRIEF-A BRI and BIS-11). Analysis of covariance compared brain donors who had impaired FAQ (i.e. 9 or higher) and BRIEF-A BRI (i.e. *T* = 65 or higher) scores versus those with unimpaired scores on PLP concentrations. Because there is no established cut-off to define clinically meaningful impairment for the BIS-11, this analysis was not performed for this scale. The estimated marginal mean diff. is reported. There is a wide age distribution of the sample and only 22 of the brain donors (of the 166 with complete clinical data) were younger than age 50 and the median age of the clinical sample was 72 (71 for the full sample). Given the sensitivity of the clinical outcomes to age, analyses were repeated and stratified by age 50.

As previously described, the MAG:PLP ratio is an indicator of *subacute* ischaemic injury from hypoperfusion.^65,67^ Because the goal of our study was to examine the long-term and not subacute effects of exposure to RHI on myelin integrity, MAG and PLP were examined separately as opposed to examining their ratio. However, given there is a precedent in the literature for MAG:PLP, the primary analytic models described above were repeated for the MAG:PLP ratio as *post hoc* analyses.

A *P*-value <0.05 defined statistical significance. Two analyses were performed for each RHI exposure metric and three for each clinical scale. Due to the limited number of outcomes, adjustment for multiple comparisons was not performed. All primary analytic models investigating RHI and clinical associations with MAG and PLP controlled for age at death, level of education (less than high school, high school/GED degree, college degree, graduate degree) and arteriolosclerosis (moderate/severe versus none/mild). Arteriolosclerosis was included as a covariate to account for small vessel disease associated with vascular risk factors. The modified ischaemic injury scale was not used due to missingness on this variable. CTE stage (none, low and high) was included as an additional covariate in models examining years of play and clinical outcomes due to its known associations with these variables. Years of football play was included as a covariate for models that examine age of first exposure to reduce the possibility that any observed effects from age of first exposure are related to it being a proxy for duration of play.

## Results

Table 1 summarizes sample characteristics. All 205 participants were male; the mean age at autopsy was 67.17 (SD = 16.78, median = 71, range = 15–90), and 22 (10.8%) were Black. Older age was associated with lower PLP (*r* = −0.16, *P* = 0.02) and MAG concentrations (r = −0.14, *P* = 0.047). MAG and PLP were not associated with education level, race, hypertension, elevated cholesterol or diabetes (*P*s > 0.10). As described above, ELISAs for MAG and PLP were performed in three batches. Analysis of variance with Tukey *post hoc* showed there were no statistically significant differences in MAG concentrations between the three batches (*P*s = 0.05, 0.09, 0.97). For PLP, one batch had higher PLP concentrations compared with the other two batches (*P* < 0.001). There were no significant differences between the other two batches (*P* = 0.52). MAG or PLP batch was not associated with age at death, years of football play, FAQ scores, BIS-11 scores or BRIEF-A BRI scores. There were differences across the MAG and PLP batches for age of first exposure. For PLP, two of the batches, including the one that had higher PLP values, had an older age of first exposure compared with the other batch (*P*s < 0.05). For MAG, two of the batches also had an older age of first exposure compared with the other batch (*P*s < 0.05).

**Table 1:** Sample demographic, athletic and medical characteristics

	Total sample (*N* = 205)	<11 Years of football play (*N* = 77)	≥11 Years of football play (*N* = 128)	*P*-value
Demographics				
Age of death, mean (SD) years	67.17 (16.78)	65.01 (19.05)	68.46 (15.18)	0.18
Race, *n* (%) Black or African American	22 (10.8)	0	22 (17.3)	<0.01
Sex, *n* (%) female	0	0	0	−
Education level, *n* (%)				0.06
Less than high school/some high school	2 (1.0)	1 (1.3)	1 (0.8)	
High school/GED	55 (26.8)	25 (32.5)	30 (23.4)	
College degree	102 (49.8)	29 (37.7)	73 (57.0)	
Graduate degree	46 (22.4)	22 (28.6)	24 (18.8)	
Athletics				
Years of football play, mean (SD)	12.62 (5.56)	7.16 (2.64)	15.90 (4.06)	<0.01
Age of first exposure to football, mean (SD)	11.73 (2.87)	12.64 (2.28)	11.18 (3.05)	<0.01
Highest level played, *n* (%)				<0.01
Youth	3 (1.5)	3 (3.9)	0	
High school	31 (15.1)	29 (37.7)	2 (1.6)	
College	65 (31.7)	31 (40.3)	34 (26.6)	
Semi-professional	9 (4.4)	4 (5.2)	5 (3.9)	
Professional	97 (47.3)	10 (13.0)	87 (68.0)	
Football primary position (at the highest level), *n* (%)				0.78
Offensive lineman	33 (16.1)	9 (11.7)	24 (18.8)	
Defensive lineman	36 (17.6)	16 (20.8)	20 (15.6)	
Linebacker	26 (12.7)	3 (3.9)	23 (18.0)	
Defensive back	12 (5.9)	1 (1.3)	11 (8.6)	
Tight end	6 (2.9)	2 (2.6)	4 (3.1)	
Quarterback	9 (4.4)	3 (3.9)	6 (4.7)	
Running back	20 (9.8)	6 (7.8)	14 (10.9)	
Wide receiver	7 (3.4)	2 (2.6)	5 (3.9)	
Special teams	2 (1.0)	2 (2.6)	0	
Other	1 (0.5)	1 (1.3)	0	
Multiple	48 (23.4)	27 (35.1)	21 (16.4)	
Unknown	5 (2.4)	5 (6.5)	0	
Other contact sport history, *n* (%)	57 (27.8)	28 (36.4)	29 (22.7)	0.03
Military history, *n* (%)	56 (33.5)	21 (32.3)	35 (34.3)	0.79
% combat exposure, *n* (%)	10 (6.0)	6 (9.2)	4 (3.9)	0.19
Cardiovascular disease, *n* (%)				
Hypertension	97 (53.0)	37 (53.6)	60 (52.6)	0.90
Diabetes	34 (18.4)	11 (15.9)	23 (19.8)	0.51
Hypercholesterolaemia	76 (42.5)	31 (46.3)	45 (40.2)	0.43
Clinical scales, mean (SD)				
FAQ	18.73 (11.37)	19.10 (11.40)	18.52 (11.39)	0.74
BRIEF-A BRI (T-score)	79.50 (18.27)	81.52 (16.47)	78.28 (19.26)	0.27
BIS-11	71.04 (16.37)	69.40 (16.12)	72.00 (16.51)	0.32

Chi-square or Fisher’s Exact Test (for binary outcomes) and independent samples *t*-test (for continuous outcomes) compared those who played <11 and 11 or more years of football. For position played, linemen were compared with all others. Higher scores on clinical scales are worse. Samples sizes were reduced for race and age of first exposure, *n* = 204; military history, *n* = 167; hypertension, *n* = 183; diabetes, *n* = 185; hypercholesterolaemia, *n* = 179; FAQ, *n* = 187; BRIEF-A BRI, *n* = 170; BIS-11, *n* = 170.

### Neuropathological correlates of MAG and PLP

As shown in Table 2, CTE was the most common neuropathological diagnosis (*n* = 151, 73.7%) and 91/151 (60.3%) had CTE without a significant co-morbid neurodegenerative disease diagnosis. Alzheimer’s disease was the second most common neurodegenerative disease diagnosis (*n* = 49, 23.9%). Other neurodegenerative diseases were less frequent. Although there were no differences between brain donors with and without CTE in MAG and PLP concentrations (*P* > 0.10), PLP levels (but not MAG) were lower among brain donors with high-stage CTE compared with those who had low-stage CTE (mean diff. = 29.68, *P* = 0.03). MAG or PLP demonstrated no statistically significant associations with Alzheimer’s disease, Lewy body disease or cerebral amyloid angiopathy (*P*s > 0.05 for all).

**Table 2:** Sample neuropathology characteristics

	Total sample (*N* = 205)	<11 Years of football play (*N* = 77)	≥11 Years of football play (*N* = 128)	*P*-value
CTE Stage, *n* (%)				<0.01
Stage 0 (no CTE)	54 (26.3)	33 (42.9)	21 (16.4)	
Stage I/II (low)	39 (9.3)	20 (26.0)	19 (14.8)	
Stage III/IV (high)	112 (54.6)	24 (31.2)	88 (68.8)	
Lewy body disease				0.87
Brainstem predominant	22 (10.7)	8 (10.4)	14 (10.9)	
Limbic (transitional)/neocortical	22 (10.7)	9 (11.7)	13 (10.2)	
Frontotemporal lobar degeneration	18 (8.8)	5 (6.5)	13 (10.2)	0.45
Motor neuron disease	3 (1.5)	0	3 (2.4)	−
Alzheimer’s disease	49 (23.9)	25 (32.5)	24 (18.8)	0.03
CERAD neuritic plaque score				0.03
No neuritic plaques	113 (55.1)	42 (54.5)	71 (55.5)	
Sparse neuritic plaques	52 (25.4)	13 (16.9)	39 (30.5)	
Moderate neuritic plaques	31 (15.1)	17 (22.1)	14 (10.9)	
Frequent neuritic plaques	9 (4.4)	5 (6.5)	4 (3.1)	
Braak stage				0.02
Stage 0	42 (20.5)	22 (28.6)	20 (15.6)	
Stage I/II	34 (16.6)	13 (16.9)	21 (16.4)	
Stage III/IV	75 (36.6)	19 (24.7)	56 (43.8)	
Stage V/VI	52 (25.4)	23 (29.9)	29 (22.7)	
Modified ischaemic injury score, mean (SD)	5.35 (3.02)	4.77 (2.75)	5.69 (3.13)	0.04
Cerebral amyloid angiopathy, *n* (%) moderate-severe	54 (26.3)	24 (31.2)	30 (23.4)	0.22
Arteriosclerosis, *n* (%) moderate-severe	125 (61.0)	46 (59.7)	79 (61.7)	0.78
Atherosclerosis, *n* (%) moderate-severe	46 (22.9)	10 (13.3)	36 (28.6)	0.01
Remote microinfarcts, *n* (%) present	48 (23.4)	13 (16.9)	35 (27.3)	0.09
Remote infarcts, *n* (%) present	32 (15.7)	8 (10.4)	24 (18.9)	0.12
White matter rarefaction, *n* (%) moderate-severe	102 (50.0)	29 (38.2)	73 (57.0)	0.01
MAG frontal, mean (SD) pg/ml	527.32 (134.58)	553.74 (139.40)	511.42 (129.56)	0.03
PLP frontal, mean (SD) pg/ml	168.72 (74.19)	181.31 (77.73)	161.15 (71.22)	0.06

Chi-square or Fisher’s Exact Test (for binary outcomes) and independent samples *t*-test (for continuous outcomes) compared those who played <11 years of football to those who played 11 or more years. Analyses that compare MAG and PLP are unadjusted in this table. As reported in the Results, there are statistically significant differences between the exposure groups for both MAG and PLP after adjusting for age at death, level of education, arteriolosclerosis and CTE stage. The modified ischaemic injury scale is a summary composite of semi-quantitative ratings of severity of hippocampal sclerosis, infarcts/lacune (absent/present), microinfarcts (absent/present), microbleeds (absent/present), laminar necrosis, arteriolosclerosis, atherosclerosis, cerebral amyloid angiopathy and white matter rarefaction (possible range: 0–17). Of the 205 brain donors, sample sizes were reduced for the following due to missing data: Braak stage, *n* = 203; modified ischaemic injury scale, *n* = 187; atherosclerosis, *n* = 201; white matter rarefaction, *n* = 204.

CTE, chronic traumatic encephalopathy; CERAD, Consortium to Establish a Registry for Alzheimer’s Disease.

Cerebrovascular disease pathologies were frequent in the sample. Among the subset with the modified ischaemic injury scale (*n* = 187), lower MAG (*r* = −0.23, *P* < 0.01) and PLP (*r* = −0.20, *P* < 0.01) concentrations were associated with higher scores on this composite (i.e. greater cerebrovascular disease). More severe arteriolosclerosis was associated with lower PLP concentrations (mean diff. = 23.47, *P* = 0.04). More severe white matter rarefaction (mean diff. = 38.79, *P* = 0.04) and atherosclerosis (mean diff. = 57.84, *P* = 0.01), as well as the presence of remote infarcts (mean diff. = 56.39, *P* = 0.03) and microinfarcts (mean diff. = 59.45, *P* = 0.01) were associated with lower MAG levels.

### Years of play and MAG and PLP

**Figure 1:**
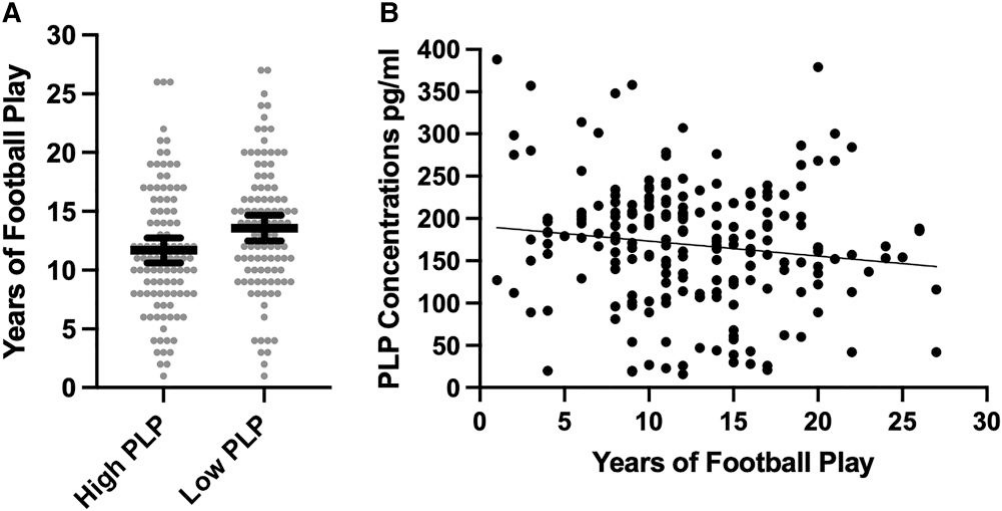
**Association between years of American football play and PLP concentrations.** (**A**) Distribution of years of American football play by PLP concentrations dichotomized into high and low based on the sample median split. The bar is the mean and the error bars are 95% CI. The figure shows unadjusted associations. Analysis of covariance controlling for age, education level, arteriolosclerosis and CTE stage showed the difference to be significant (*F* = 6.90, mean diff. = 1.79, 95% CI = 0.45–3.13, *P* = 0.01). (**B**) Scatter plot of the association between years of American football play and PLP as a continuous variable. The figure shows unadjusted associations. Multivariable linear regression models showed that more years of football play was associated with lower PLP concentrations (unstandardized beta = −2.45, 95% CI = −4.52, −0.38, *t* = −2.34, *P* = 0.02), adjusting for age at death, level of education, arteriolosclerosis and CTE stage.

Multivariable linear regression models showed that more years of football play was associated with lower PLP concentrations (unstandardized beta = −2.45, 95% CI [−4.52, −0.38], *P* = 0.02), adjusting for age at death, level of education, arteriolosclerosis and CTE stage. Greater years of football play was associated with increased odds of having low (i.e. less than the median) PLP concentrations [odds ratio (OR) = 1.08, 95% CI [1.02, 1.15], *P* = 0.01]. Those with low PLP concentrations played approximately two more years of football compared with those who had high PLP concentrations (mean diff. = 1.79, *P* = 0.01) (see Fig. 1). Years of football play was not associated with MAG concentrations as a continuous variable (unstandardized beta = −3.00, 95% CI [−6.80, 0.80], *P* = 0.12) or when examined dichotomized into high and low (OR = 1.06, 95% CI [0.99, 1.12], *P* = 0.07).

#### Cut-off sensitivity analyses

Compared with those who played <11 years of football (*n* = 78), those who played 11 or more years (*n* = 128) were more likely to be Black. However, no other differences in demographic, medical or clinical characteristics existed between the RHI duration groups (Table 1). Regarding neuropathology, those who played 11 or more years of football had higher rates of CTE, more severe CTE, more severe atherosclerosis and white matter rarefaction and lower rates of Alzheimer disease (Table 2). Compared with those who played <11 years of football, those who played 11 or more years of football had lower MAG (mean diff. = 46.00, 95% CI [5.32, 86.69], *P* = 0.03) and PLP concentrations (mean diff. = 24.72, 95% CI [2.40, 47.05], *P* = 0.03).

### Age of first exposure and MAG and PLP

Younger age of first exposure to football was associated with lower PLP concentrations (unstandardized beta = 4.35, 95% CI [0.25, 8.45], *P* = 0.04). Association between younger age of first exposure and increased odds for having low PLP concentrations was trending (OR = 0.89, 95% CI [0.79, 1.00], *P* = 0.05). Age of first exposure was not associated with MAG concentrations as a continuous variable (unstandardized beta = 3.63, 95% CI [−3.92, 11.19], *P* = 0.34) or when examined dichotomized into high and low (OR = 0.98, 95% CI [0.87, 1.10], *P* = 0.68).

### MAG and PLP: associations with clinical function

**Figure 2:**
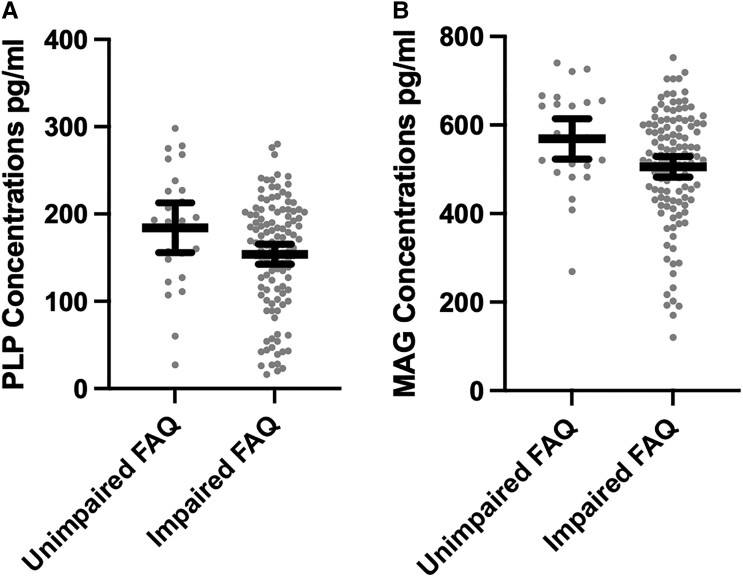
**Distribution of PLP and MAG concentrations by FAQ Status among older brain donors.** The *x*-axis for both is the FAQ dichotomized into impaired (score of 9 or higher) and unimpaired (score <9). The bar is the mean and the error bars are 95% CI. The sample is restricted brain donors who were 50 years or older at the time of death. The figure shows unadjusted associations. Analysis of covariance controlling for age, education level, arteriolosclerosis and CTE stage show differences to be statistically significant: (**A**) *F* = 4.48, mean diff. = 33.71, *P* = 0.036 and (**B**) *F* = 4.43, mean diff. = 66.58, *P* = 0.037.

Table 3 summarizes the clinical scale regression models. Of the 166 brain donors with complete data on the clinical scales, 126 (75.9%) brain donors were reported by an informant to be functionally impaired (i.e. FAQ score of 9 or higher) and 126 (75.9%) were reported to have had clinically meaningful neurobehavioural dysregulation (BRIEF-A BRI *T*-score ≥65). MAG and PLP (as continuous variables or dichotomized into high versus low) were *not* associated with the FAQ, BRIEF-A BRI or the BIS-11 (*P*s > 0.05). The young brain donors might have been suppressing effects for clinical scales, particularly given age is associated with MAG and PLP (shown above) as well as the FAQ (*r* = 0.61, *P* < 0.001) and the BIS-11 (*r* = −0.23, *P* < 0.01). Clinical scale models were repeated after restricting the sample to brain donors who were 50 years or older. In this subgroup, lower PLP and MAG concentrations were associated with higher FAQ scores (i.e. worse functioning) (Table 3). As shown in Fig. 2, brain donors who had impaired FAQ scores had lower PLP (mean diff. = 33.71, *P* = 0.036) and MAG (mean diff. = 66.58, *P* = 0.037) concentrations compared with brain donors who had unimpaired scores (i.e. FAQ score < 9). Lower MAG levels were also associated with higher BIS-11 scores (i.e. greater impulsivity). There continued to be no association with the BRIEF-A BRI.

**Table 3:** Associations between MAG and PLP and clinical scales

	Total sample (*N* = 166)											
	FAQ				BIS-11				BRIEF-A BRI			
	Unstd. beta	95% CI	Std. beta	*P*	Unstd. beta	95% CI	Std. beta	*P*	Unstd. beta	95% CI	Std. beta	*P*
MAG (pg/ml)	−0.01	−0.02, 0.00	−0.09	0.17	−0.01	−0.03, 0.01	−0.10	0.19	0.01	−0.01, 0.03	0.09	0.28
PLP (pg/ml)	−0.02	−0.04, 0.01	−0.09	0.15	0.00	−0.04, 0.04	−0.001	0.99	0.02	−0.02, 0.06	0.07	0.36
	≥50 years (*n* = 144)											
MAG (pg/ml)	−0.01	−0.03, −0.002	−0.17	**0**.**02**	−0.02	−0.04, −0.0003	−0.16	**0**.**047**	0.002	−0.02, 0.03	0.01	0.88
PLP (pg/ml)	−0.02	−0.047, −0.001	−0.15	**0**.**04**	−0.01	−0.05, 0.03	−0.04	0.63	0.001	−0.04, 0.05	0.01	0.96

Multivariable linear regressions examined the association between MAG and PLP and each clinical scale controlling for age, education level, arteriolosclerosis and CTE stage. Higher scores reflect greater functional difficulties on the FAQ, greater impulsivity on the BIS-11, and greater behavioural dysregulation on the BRIEF-A BRI.

FAQ, Functional Activities Questionnaire; BIS-11, Barratt Impulsiveness Scale-11; BRIEF-A BRI, Behavior Rating Inventory of Executive Functions, Adult Version (BRIEF-A) Behavioral Regulation Index (BRI); unstad, unstandardized; std, standardized.

### *Post hoc* analyses: MAG:PLP ratio

Linear regressions showed that years of football play was not associated with MAG:PLP, controlling for age, education, arteriolosclerosis and CTE stage (*P* = 0.10). There was a statistically significant association between age of first exposure and MAG:PLP (unstandardized beta = −0.15, 95% CI [−0.28, −0.01], *P* = 0.04). However, the negative coefficient was in the opposite than expected direction, i.e. younger age of first exposure to football was associated with higher MAG:PLP values. MAG:PLP was not associated with any of the clinical scales in the full sample or among those who were 50 years or older (*P*s > 0.05 for all).

## Discussion

In this study, we examined the pathological, clinical and risk correlates of myelin integrity using immunoassays for MAG and PLP in frontal cortex white matter from brain donors who played American football. Results showed that lower MAG and PLP were associated with cerebrovascular disease pathologies. Lower PLP was associated with more severe CTE, but MAG and PLP did not correlate with other neurodegenerative disease pathologies. Proxies of exposure to RHI, including years of football play and age of first exposure to football, emerged as significant correlates of MAG and PLP independent of age, education, arteriolosclerosis and CTE pathology. Among older brain donors (i.e. 50+ years), lower MAG and/or PLP were associated with greater informant-reported functional and impulse control difficulties. Our findings suggest that decreased myelin proteins are a consequence of exposure to RHI and may be a potential contributor to clinical symptoms.

Greater years of football play was associated with lower PLP. Compared with those who played fewer than 11 years of football, those who played 11 years or more had lower MAG and PLP. Years of American football, a proxy for the duration of exposure to RHI, has dose–response relationships with CTE status, with 11 years or more of play maximizing risk.^14^ Years of exposure to RHI is associated with other neurodegenerative and non-neurodegenerative disease pathologies.^32,34,54,85–87^ Expansive axonal and myelin degradation has long been described in autopsy case series of CTE.^52,54,88^ Empirical studies have also shown more years of football play is associated with greater severity of white matter rarefaction in 180 older brain donors with CTE from the UNITE brain bank.^54^*In vivo* magnetic resonance imaging studies also demonstrate later-life white matter and related vascular changes from RHI^17,46,48,55,89–91^; however, the literature is inconsistent.^92–94^ The lack of granular myelin-specific assessments has limited our understanding of the association between RHI and myelin degeneration.

The present study is the first to directly test RHI and myelin associations using immunoassays of MAG and PLP in the frontal cortex tissue of 205 brain donors. MAG and PLP are produced in oligodendrocytes and delivered to the myelin sheath.^59,60^ They contribute to the structure, function and maintenance of the myelin and reductions of these proteins typically reflect myelin loss or degeneration. These proteins might be reduced as a result of damage from RHI. Oligodendrocytes are severely affected in the white matter in CTE^53^ and MAG, an oligodendrocyte-specific protein, is decreased in the white matter in CTE.^69^ Our observations of the associations between years of football play and MAG and PLP support the hypothesis of MAG and PLP breakdown from RHI. Notably, this association was independent of age, CTE and arteriolosclerosis, but the effect was small and significant heterogeneity was present (Fig. 1). Although CTE severity was associated with lower PLP, the effect size was small and PLP was not associated with CTE status. Case-control studies in Alzheimer’s disease also show mixed results, including those that show no reductions in MAG and PLP (or MAG:PLP)^63–65^ and others that found reductions in MAG:PLP in cortical grey matter in early Alzheimer’s disease and vascular dementia.^60,62^ In Alzheimer’s disease and ageing, MAG and PLP seem most likely to be affected by the presence of small vessel disease.^60,64,65^ In people exposed to RHI, myelin degeneration could represent a separate (e.g. from CTE, ageing and vascular risk factors) pathological consequence of RHI that is parallel to other RHI and non-RHI related pathologies.

Younger age of first exposure to football was associated with lower PLP but not MAG, after controlling for age, education, years of football play and arteriolosclerosis. Younger age of first exposure has been linked with white matter alterations of the anterior corpus callosum in former National Football League players.^17^ A recent study found younger age of first exposure was associated with a greater burden of white matter hyperintensities on FLAIR MRI in older but not younger former college and professional football players.^95^ Participation in football as a youth and adolescent can result in acute white matter changes on diffusion tensor imaging.^39,96,97^ Our present findings extend this evidence by showing that younger age of first exposure to RHI is associated with lower PLP concentrations, which may be a result of interfering with normal white matter development where myelination is known to continue through age 20 and beyond. Notably, age of first exposure is not associated with CTE status and thus the age of first exposure–white matter association is likely to be independent of tau or neurodegeneration, whereas the relationship between years of play and white matter is more complex given the close association between duration of play and CTE pathology.^16^ However, it is important to interpret our findings in the context of the broader literature on age of first exposure. Specifically, research shows no association between age of first exposure and neurobehavioural function in young healthy amateur contact sport athletes,^27,28,98^ whereas the literature is mixed in older samples of former American football players.^99,100^

Among older brain donors, lower MAG and PLP correlated with greater informant-reported functional difficulties and lower MAG was associated with informant-reported impulsiveness. A constellation of cognitive, mood and behavioural symptoms have been reported in individuals exposed to RHI.^10,11,30,82,101,102^ However, inconsistencies across studies exist.^102–104^ The 2021 traumatic encephalopathy syndrome research diagnostic criteria,^30^ the purported clinical syndrome of CTE, describes the core clinical features of CTE to include cognitive impairment and neurobehavioural dysregulation including but not limited to impulsivity. The p-tau of CTE has been associated with informant-reported cognitive but not behavioural/mood symptoms.^31^*In vivo* studies have also failed to show an association between tau and symptoms.^105,106^ Symptoms of neurobehavioural dysregulation might be better accounted for by non-tau neuropathologies, specifically of the white matter. White matter rarefaction predicted increased odds for dementia to a similar extent as p-tau among older brain donors with CTE.^54^*In vivo* MRI studies document the contribution of white matter injury to neuropsychiatric, in addition to cognitive symptoms, in people exposed to RHI.^46,48,51^ In Alzheimer’s disease, pathologies of the white matter have been shown to contribute to neuropsychiatric symptoms including behavioural dysregulation.^107–109^ Overall, the aetiology of symptoms associated with exposure to RHI is likely to be multifactorial. If white matter or other pathologies better explain symptoms of neurobehavioural dysregulation in people exposed to RHI compared with tau, this would have important implications for revisions of the traumatic encephalopathy syndrome research diagnostic criteria.

Declines in the MAG:PLP ratio reflect acute cerebral hypoperfusion in the tissue.^59,60,65,67^ The ratio has been the focus of studies investigating the contribution of hypoperfusion and resulting ischaemia to the pathogenesis of Alzheimer’s disease and related dementias.^60,63–65^ Our *post hoc* analyses showed minimal associations with the MAG:PLP ratio. This was not unexpected as our objectives and *a priori* hypotheses targeted MAG and PLP separately (as opposed to their ratio) as a means to elucidate the late, rather than subacute, effects of RHI on myelin integrity.

The findings of our study have some important limitations worth noting. First, we included a convenience sample of symptomatic brain donors who played American football, allowing for a sample with similarities in athletic and exposure to RHI characteristics. The generalizability of our findings is thus limited to male football players who present to a clinic due to concerns related to their cognitive, mood and behavioural status. Relatedly, selection biases associated with brain donation must be considered as individuals who have exposure to RHI and are symptomatic are more likely to donate to the UNITE brain bank. However, such selection does not negate valid RHI-CTE estimates.^14,110^ Additionally, immunoassays for MAG and PLP were done on the white matter underlying the frontal cortex as this region is commonly affected by exposure to RHI and is the initial location of tau pathology in CTE. Examination of other regions will inform specificity. Moreover, the inclusion of RHI naïve brain donors and other neurodegenerative disease comparison groups will also be important to understand the specificity of the present findings, as well as strengthen inferences regarding the observed RHI–myelin associations. An NINDS-funded U54 effort is currently underway at our centre that is harmonizing the brain bank from this study with several other brain banks not focused on RHI to ascertain non-RHI controls. Once there are sufficient sample sizes, we will compare MAG and PLP concentrations, among other pathologies, between RHI and age-matched non-RHI brain donors. Finally, our clinical scales were completed by informants of brain donors, and therefore, estimates might be inaccurate due to biases from recall and subjectivity. Prospective studies with clinical and cognitive characterization during life among people who agree to brain donation are needed for validation.

## Conclusions

More years of football play and younger age of first exposure to football were associated with decreased myelin proteins as measured by immunoassays for MAG and PLP in the frontal cortex tissue of 205 brain donors. From a clinical standpoint, both PLP and MAG were associated with greater informant-reported functional and impulse control difficulties. Myelin degeneration is another potential pathological consequence of RHI that might contribute to the manifestation of clinical symptoms. Further characterization of the pathologies that arise from RHI, including those of the white matter, and their relative contribution to objectively defined clinical and cognitive symptoms will inform future iterations of the traumatic encephalopathy syndrome research diagnostic criteria, as well as treatment and preventative targets in this vulnerable population.

## Data Availability

Data elements from the Understanding Neurologic Injury and Traumatic Encephalopathy study are available from the publicly available FITBIR data set. Other raw data from this study are available upon reasonable request to the corresponding author.

## Abbreviations

BIS-11 =: Barratt Impulsiveness Scale-11
BRI =: Behavioral Regulation Index
BRIEF-A =: Behavior Rating Inventory of Executive Function-Adult version
CTE =: chronic traumatic encephalopathy
FAQ =: Functional Activities Questionnaire
MAG =: myelin-associated glycoprotein
PLP =: proteolipid protein 1
P-tau =: hyper-phosphorylated tau
RHI =: repetitive head impacts
